# Relieving pandemic-related psychological distress: Key protective factors against mental health impairment

**DOI:** 10.1192/j.eurpsy.2023.2357

**Published:** 2023-07-19

**Authors:** D. Alonzo, M. Popescu, P. Zubaroglu

**Affiliations:** 1 Fordham University, West Harrison, United States

## Abstract

**Introduction:**

Despite studies pointing to the important role of relational and community factors in influencing mental health during times of crises such as the current pandemic, little research has examined protective factors at the relational/community level that serve protective factors against mental health impairment in response to the current pandemic.

**Objectives:**

This study aims to address this gap and examines protective factors against adverse mental health consequences related to the pandemic at the relational and community levels among individuals residing in high-risk marginalized low-resourced settings in Guatemala at one and a half years post onset of the pandemic

**Methods:**

Telephone surveys were administered to 100 participants to assess sociodemographic characteristics, psychosocial functioning, and protective factors (interpersonal support, psychoeducation, community resources, and adaptive coping) against psychological distress. (anxiety, depression, stress, burnout). Multiple linear regressions were used to examine predictors of mental health impairment.

**Results:**

Our findings demonstrate that only psychoeducation serves a protective factor against psychological distress.Interpersonal support was found to predict increased levels of anxiety and depression and adaptive coping was found to predict increased levels of anxiety, depression, and burnout. No significant relationship between community resources and any type of mental health impairment was found.

**Conclusions:**

Public mental health efforts should capitalize on the effectiveness of psychoeducation to promote strategies for managing symptoms of psychological distress as well as providing information regarding resources and services. In the context of complex emergencies that have an immediate effect on already scarce resources at a personal, community, and institutional levels, psychoeducation has the advantage of a low-cost intervention, easily transferable between communities, providing immediate support as well as sustainability over time.

**Disclosure of Interest:**

None Declared

